# *Porphyromonas gingivalis* Outer Membrane Vesicles Mediate Coaggregation and Piggybacking of *Treponema denticola* and *Lachnoanaerobaculum saburreum*

**DOI:** 10.1155/2013/305476

**Published:** 2013-01-13

**Authors:** Daniel Grenier

**Affiliations:** Groupe de Recherche en Écologie Buccale, Faculté de Médecine Dentaire, Université Laval, 2420 de la Terrasse, Quebec City, QC, Canada G1V 0A6

## Abstract

*Porphyromonas gingivalis* sheds outer membrane vesicles that contain several virulence factors, including adhesins. In this study, we investigated the ability of *P. gingivalis* outer membrane vesicles to mediate the coaggregation and piggybacking of *Treponema denticola* and *Lachnoanaerobaculum saburreum*. Marked coaggregation between *T. denticola* and *L. saburreum* occurred in the presence of *P. gingivalis* outer membrane vesicles. Sucrose was an effective chemoattractant for the motile species *T. denticola*. The addition of outer membrane vesicles to a mixture of *T. denticola* and *L. saburreum* significantly increased the number of nonmotile bacteria that migrated into a sucrose-filled capillary tube immersed in the bacterial mixture. Under optimal conditions, the number of nonmotile *L. saburreum* in the capillary tube increased approximately 5-fold, whereas no increase occurred when boiled vesicles were used. This study showed that *P. gingivalis* outer membrane vesicles mediate coaggregation between *T. denticola* and *L. saburreum* and that nonmotile bacteria can be translocated by piggybacking on spirochetes.

## 1. Introduction

Outer membrane vesicles (10–300 nm in diameter) are naturally shed from the surfaces of Gram-negative bacteria. While they are produced under a variety of growth conditions, shedding appears to occur mainly in response to a stress [1]. The exact mechanism of vesiculation is not fully understood but seems to result from the budding of the bacterial envelope in areas where lipoprotein links between the outer membrane and the peptidoglycan are broken or missing [2]. *Porphyromonas gingivalis*, a major pathogen in chronic periodontitis, is known to shed large numbers of outer membrane vesicles [3] which have been reported to increase when *P. gingivalis* is grown in hemin-restricted conditions [4]. Since several determinants of *P. gingivalis* virulence such as adhesins and proteinases are surface associated, the shedding of outer membrane vesicles may contribute to the pathogenic process of periodontitis. Biologically active substances are present in larger amounts in outer membrane vesicles than in whole bacterial cells, likely due to the higher surface : volume ratio of the vesicles [3]. Previous studies have reported that *P. gingivalis* outer membrane vesicles can adhere to bacteria [5–8], hydroxyapatite surfaces [7], and host cells [6, 8]. Inagaki et al. [8] provided evidence for a role of *P. gingivalis* outer membrane vesicles in periodontal tissue invasion. More specifically, they showed that outer membrane vesicles can mediate the adherence to and invasion of oral epithelial cells by* Tannerella forsythia*.

*Treponema denticola* and some other spirochetes play a major role in the development and progression of periodontitis [9]. These periplasmic flagellated bacteria appear to preferentially colonize the apical area of the periodontal pocket [10, 11]. The chemotactic response of spirochetes is important in the microbial ecology of the subgingival crevice [12, 13]. Two methyl-accepting chemotaxis genes (*dmcA* and *dmcB*) have been identified and characterized in *T. denticola *[14, 15]. Lux et al. [16] suggested that chemotaxis is involved in periodontal tissue invasion since a chemotaxis mutant was unable to penetrate the tissue despite being motile. The colonization of the apical areas by nonmotile bacteria may be mediated by piggybacking on motile bacteria such as spirochetes, a phenomenon by which nonmotile bacteria attached to motile bacteria are translocated to another area. In this study, we investigated the capacity of *P. gingivalis* outer membrane vesicles to mediate piggybacking using a model mixture of *T. denticola* and* Lachnoanaerobaculum saburreum* (formerly, *Eubacterium saburreum*), a Gram-positive bacterium found in dental biofilms that has been associated with endodontic infections [17, 18].

## 2. Materials and Methods

### 2.1. Bacterial Strains and Growth Conditions

*T. denticola* ATCC 35405 was grown in New Oral Spirochete (NOS) medium [19], *L. saburreum* 162.4 in Brain Heart Infusion broth (Becton, Dickinson, Sparks, MD, USA), and *P. gingivalis* ATCC 33277 in Brain Heart Infusion broth supplemented with 10 *μ*g/mL of hemin and 1 *μ*g/mL of vitamin K. The cultures were incubated in an anaerobic chamber (N_2_-H_2_-CO_2_/80 : 10 : 10) at 37°C.

### 2.2. Preparation of Outer Membrane Vesicles

*P. gingivalis* outer membrane vesicles were isolated from 2-day-old cultures by ultrafiltration and ultracentrifugation, as previously described [6]. The vesicle preparations were freeze dried. The vesicles (5 mg/mL) were resuspended in phosphate-buffered saline (PBS, pH 7.2) prior to use and were lightly sonicated to break up large aggregates.

### 2.3. Coaggregation Assay

*T. denticola *and *L. saburreum* cells were washed twice by centrifugation (5000 ×g for 15 min) and were resuspended in PBS to an optical density of 0.5 at 660 nm. Equal volumes (150 *μ*L) of both bacterial species together with 10 *μ*L of either PBS or the outer membrane vesicle preparation were mixed in 12 × 75 mm round-bottom test tubes and were incubated at 30°C for 5 min on a rocking platform shaker (100 rpm). Coaggregation was scored using a visual rating scale of 0 to 4+ as previously described (see Table 1 for details) [20]. The effect of outer membrane vesicles on aggregation of the two bacterial species taken individually was also tested.

### 2.4. Capillary Chemotaxis Assay of *T. denticola*

The chemotaxis assay was based on the procedure described by Ruby et al. [12]. Briefly, *T. denticola* cells from 2-day-old cultures were harvested by low speed centrifugation (2000 ×g for 10 min) and were suspended in prereduced chemotaxis buffer (0.15 M NaCl, 10 mM NaH_2_PO_4_, pH 7.6) containing 0.5% methylcellulose to an optical density of 0.25 at 660 nm. The cell suspension (200 *μ*L) was placed in a 1.5 mL microtube, and the tip of a capillary tube (1 *μ*L) filled with potential chemoattractants was immersed (1 mm deep) in the bacterial suspension. The capillary tube was held in place by inserting it through a hole in the microtube cap. Potential chemoattractants prepared in chemotaxis buffer included 0.02% (w/v) cellobiose, fructose, glucose, lactose, sucrose, mannitol, arginine, and cysteine. After a 60 min incubation at 37°C, the number of spirochetes that had migrated into the capillary tube was evaluated using a phase contrast microscope at a magnification of 1000x. Chemotaxis (average of 5 assays) was expressed as follows: −, ≤1 bacterium/microscopic field; +, 2–5 bacteria/field; ++, 6–15 bacteria/field; +++, ≥16 bacteria/field.

### 2.5. Piggyback Assay of* T. denticola *and* L. saburreum*

*T. denticola *and *L. saburreum* cells from 2-day-old cultures were resuspended in pre-reduced chemotaxis buffer containing 0.5% methylcellulose to an optical density of 0.25 at 660 nm, and 100 *μ*L of each suspension alone (plus 100 *μ*L of PBS) or a mixture of 100 *μ*L of each suspension were placed in 1.5 mL microtubes. *P. gingivalis *vesicles (10 *μ*L of a 5 mg/mL suspension) or PBS (10 *μ*L, control) were added to the microtubes, and the mixtures were vortexed prior to inserting the capillary tubes filled with chemotaxis buffer containing 0.5% methylcellulose and 0.02% sucrose into the bacterial suspensions. After a 60 min incubation at 37°C, the capillary tubes were removed and were examined using a phase contrast microscope at a magnification of 1000x. The numbers of *T. denticola *and *L. saburreum *cells in the capillary tubes were determined using a Petroff-Hausser counting chamber according to the manufacturer's instructions. All experiments were performed 10 times, and the means ± standard deviation (SD) were calculated. Experimental data were analyzed for significance using Student's *t*-test.

## 3. Results and Discussion

In the absence of *P. gingivalis* outer membrane vesicles, *T. denticola* and* L. saburreum* did not coaggregate (Table 1). However, when vesicles were added, marked coaggregation occurred. The ability of outer membrane vesicles to mediate coaggregation between *T. denticola* and* L. saburreum* was dose dependent. Moreover, a heat treatment of the outer membrane vesicles resulted in an absence of coaggregation suggesting that the adhesins involved possess a proteinaceous nature. The vesicles had only a minor effect on aggregation of the two species taken individually. *P. gingivalis* outer membrane vesicles have previously been shown to mediate coaggregation between *Capnocytophaga ochracea* and *L. saburreum* [6].

Several of the carbohydrates tested for their potential as chemoattractants (see Table 2) attracted large numbers of *T. denticola*, with sucrose eliciting the strongest response. Cellobiose, fructose, glucose, and, to a lesser extent, lactose and mannitol also acted as chemoattractants. Sorbitol and the two amino acids (arginine and cysteine) had no effect. These results were in agreement with those of Mayo et al. [21], who reported that *T*. *denticola* was attracted to 11 of the 13 carbohydrates they tested, including sucrose. Investigations of the chemotactic behavior of *Spirochaeta aurantia*, a spirochete found in sewage samples, showed that it is attracted to a variety of sugars, which serve as energy sources for its growth [22].

Since sucrose elicited the strongest response, it was used as the chemoattractant in the piggyback assay. When the two bacterial species were mixed in the absence of vesicles, 67 ± 22 × 10^3^ and 4 ± 3 × 10^3^ *T. denticola* and *L. saburreum* cells, respectively, were observed in the capillary tube (Table 3). Under these conditions, neither species had an effect on the migration of the other.

**Figure 1:**
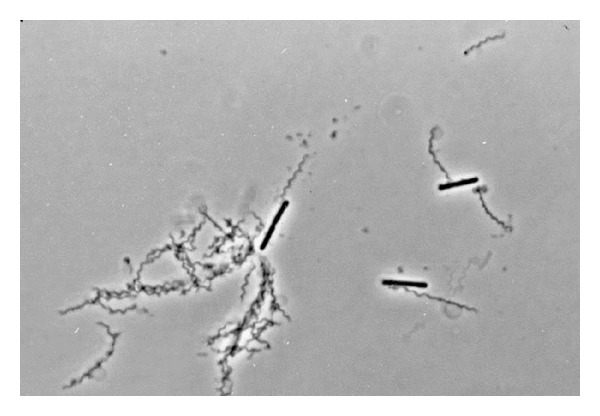
Phase contrast micrograph showing *L. saburreum *bound to *T. denticola *in the presence of *P. gingivalis *outer membrane vesicles following the piggyback assay. Magnification: 1000x.

The addition of *P. gingivalis* outer membrane vesicles to the mixture of *T. denticola* and *L. saburreum* significantly increased the number of *L. saburreum* cells observed in the capillary tube fivefold (*P* < 0.001) (Table 3). When the incubation period of the mixture was extended to 2 h, no increase in the migration of either of the two species was observed, possibly due to a gradual loss of spirochete motility. The phase contrast examination revealed that most of the *L. saburreum *cells were attached to spirochetes (Figure 1), either at the end or the center of the *T. denticola *cells. The addition of five times more vesicles resulted in the formation of large coaggregates and reduced the migration of both bacterial species into the capillary tube (data not shown). When boiled vesicles were added to the mixture of *T. denticola* and *L. saburreum*, the number of *L. saburreum* cells observed in the capillary tube was similar to that of the control (no vesicles), suggesting that a proteinaceous component on the surface of the vesicles may be involved in the attachment. The vesicle-mediated piggybacking mechanism reported here is unlikely to be limited to *T*. *denticola* and *L. saburreum*. Other motile and nonmotile bacteria may well behave in a similar fashion.

Outer membrane vesicles produced by Gram-negative periodontopathogens, including *P. gingivalis*, may act as bridges in biofilms, providing an environment that is resistant to antibacterial agents [2]. In this study, we provided evidence for a new function of bacterial outer membrane vesicles. We showed that *P. gingivalis* vesicles can mediate the attachment of a nonmotile bacterium (*L. saburreum*) to a motile bacterium (*T. denticola*) and modulate the piggybacking phenomenon. This observation points to a new mechanism by which outer membrane vesicles may play a role in the ecology of subgingival sites. The role of *P. gingivalis* vesicles in mediating the piggybacking phenomenon may be important for the migration of bacteria to the apical area of subgingival sites where strictly anaerobic conditions are found. Motile spirochetes covered with *P. gingivalis* outer membrane vesicles could increase the concentration of nonmotile bacteria in deep periodontal pockets, favoring tissue destruction and toxic reactions.

## Figures and Tables

**Table 1:** *P. gingivalis *outer membrane vesicle-induced coaggregation between *T. denticola *and *L. saburreum*.

Amount of outer membrane vesicles (*μ*g/mL)*	Coaggregation score^†^		
	*T. denticola *	*L. saburreum *	*T. denticola + L. saburreum *
0	0	0	0
25	0	0	0
50	0	0	1+
100	0	0	1+
250	0	0	1+
500	1	0	3+
500 (100°C/5 min)	0	0	0

*Final concentration in the assay mixture.

^†^0: no visible coaggregates in the cell suspension; 1+: small uniform coaggregates in the cell suspension; 2+: definite coaggregates easily seen but suspension remained turbid; 3+: large coaggregates that settled rapidly, leaving some turbidity in the supernatant fluid; 4+: clear supernatant fluid and large coaggregates that settled immediately.

**Table 2:** Chemotaxis of *T. denticola *associated with various compounds.

Compound*	Chemotaxis^†^
None	−
Cellobiose	++
Fructose	++
Glucose	++
Lactose	+
Sucrose	+++
Mannitol	+
Sorbitol	−
Arginine	−
Cysteine	−

*All compounds were used at 0.02% (w/v).

^†^As determined by phase-contrast microscopy at a magnification of 1000X. Chemotaxis was expressed as follows: −, ≤1 bacterium/microscopic field; +, 2–5 bacteria/field; ++, 6–15 bacteria/field; +++, ≥16 bacteria/field.

**Table 3:** Effect of *P. gingivalis *outer membrane vesicles on the piggybacking of *L. saburreum* on *T*. *denticola*.

	Mean of cells ± S.D. (×10^3^)	
Assay	Number of bacteria in capillary tube*	
	*T. denticola *	*E. saburreum *
*T. denticola* alone	71 ± 13 × 10^3^	0
*L. saburreum* alone	0	3 ± 2 × 10^3^
*T. denticola* + *L. saburreum* (without vesicles)	66 ± 22 × 10^3^	4 ± 3 × 10^3^
*T. denticola* + *L. saburreum* (with vesicles)	84 ± 26 × 10^3^	20 ± 7 × 10^3†^

∗ Determined using a Petroff-Hausser counting chamber.

^†^Significant increase (*P* < 0.001) in the number of *L. saburreum *cells in the capillary tube.
